# Macrophage migration inhibitory factor promoter polymorphisms are associated with disease activity in rheumatoid arthritis patients from Southern Mexico

**DOI:** 10.1002/mgg3.1037

**Published:** 2019-11-07

**Authors:** Guillermo Santoscoy‐Ascencio, Christian Johana Baños‐Hernández, José Eduardo Navarro‐Zarza, Jorge Hernández‐Bello, Richard Bucala, Andres López‐Quintero, Emmanuel Valdés‐Alvarado, Isela Parra‐Rojas, Berenice Illades‐Aguiar, José Francisco Muñoz‐Valle

**Affiliations:** ^1^ Instituto de Investigación en Ciencias Biomédicas Centro Universitario de Ciencias de la Salud Universidad de Guadalajara Guadalajara Jalisco Mexico; ^2^ Departamento de Biología Molecular Unidad de Patología Clínica Guadalajara Jalisco Mexico; ^3^ Facultad de Ciencias Químico‐Biológicas Universidad Autónoma de Guerrero Chilpancingo de los Bravo, Guerrero Mexico; ^4^ Departamento de Medicina Interna‐Reumatología Hospital General de Chilpancingo Dr. Raymundo Abarca Alarcón Chilpancingo de los Bravo, Guerrero Mexico; ^5^ Instituto Transdisciplinar de Investigación y Servicios Universidad de Guadalajara Zapopan Mexico; ^6^ Department of Medicine/Section of Rheumatology Yale University School of Medicine New Haven CT USA

**Keywords:** DAS28, genetic susceptibility, MIF, polymorphisms, rheumatoid arthritis

## Abstract

**Background:**

Macrophage migration inhibitory factor (MIF) is a cytokine capable of stimulating inflammatory cytokine and matrix metalloproteinase production from macrophages and synovial fibroblasts, which leads to persistent inflammation and bone degradation, two of the major pathological processes in rheumatoid arthritis (RA). The aim of this study was to evaluate the association of *MIF* promoter polymorphisms (−794CATT_5‐8_ *rs5844572* and −173G > C, *rs755622*), circulating MIF levels, and mRNA expression with RA susceptibility and disease activity.

**Methods:**

A case–control study was conducted in 200 RA patients and 200 control subjects (CS) from Southern Mexico. Genotyping was performed by conventional PCR and PCR‐RFLP methods. *MIF* mRNA expression was quantified by real‐time PCR and MIF serum levels were determined by an ELISA kit.

**Results:**

The 7,7 (−794CATT_5‐8_) and −173CC (−173G > C) genotypes were associated with higher disease activity in RA patients. MIF serum levels were increased, and *MIF* mRNA expression was reduced in RA patients as compared to CS. In addition, RA patients with moderate disease activity had higher MIF levels than those with low disease activity. The −794CATT_5‐8_ and −173G > C *MIF* polymorphisms were not associated with RA susceptibility.

**Conclusion:**

These results suggest an important role of *MIF* polymorphisms and MIF serum levels with disease activity in RA.

## Graphical abstract

MIF −794CATT5‐8 and −173G > C polymorphisms are not associated with RA susceptibility risk in a southern Mexican population. The 7,7 (−794CATT5‐8) and GC (−173G > C) genotypes are associated with high‐ and low disease activity, respectively. Higher MIF serum levels are associated with moderate disease activity in RA patients.

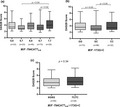

## 1 INTRODUCTION

Rheumatoid arthritis (RA) is a chronic autoimmune inflammatory disease characterized by production of autoantibodies [rheumatoid factor (RF) and anti‐citrullinated protein antibodies (ACPA)] and joint damage due to persistent synovial inflammation and invasion into adjacent articular structures, leading to irreversible cartilage damage and bone destruction (Smolen et al., 2018).

The immunopathology of RA is characterized by the interaction of multiple mediators, among the most important are cytokines, which promote autoimmunity, maintain chronic inflammatory synovitis, and drive adjacent joint tissue destruction (McInnes & Schett, 2007). Macrophage migration inhibitory factor (MIF) is an upstream immunoregulatory and potent inflammatory cytokine that is expressed by several cell types (Calandra et al., 1995; Morand, Leech, & Bernhagen, 2006). In RA, MIF is elevated in the synovium and plasma and correlates with disease activity (Llamas‐Covarrubias et al., 2013, 2012; Morand et al., 2002; Radstake et al., 2005).

MIF drives synovial macrophage release of cytokines and prostaglandins, induces the expression of disease‐promoting cytokines, regulates hypercellularity, and is required for leukocyte trafficking into the joint (Morand et al., 2006); thus, MIF is a key cytokine in diseases characterized by a disordered immune‐inflammatory response, such as RA (Morand et al., 2006).

Two polymorphisms have been described within *MIF* gene (OMIM: 153,620), a functional tetranucleotide CATT repeat located at position −794 and a G to C transversion at position −173 of the *MIF* promoter (Baugh et al., 2002; Donn, Shelley, Ollier, Thomson, & British Paediatric Rheumatology Study Group, 2001) these two variants are found in linkage disequilibrium. High expression *MIF* alleles have been associated with several autoimmune diseases, including RA (Bae & Lee, 2018; De la Cruz‐Mosso et al., 2014; Donn et al., 2002, 2001; Martínez et al., 2007; Morales‐Zambrano et al., 2014; Sreih et al., 2011, 2018).

It has been reported that *MIF* expression increases with CATT repeat number (Baugh et al., 2002), and high‐expression alleles (CATT_6‐8_) are associated with an increased risk for RA and more severe joint erosion among RA patients; whereas the low expression allele (CATT_5_) has been associated with a less aggressive rheumatoid disease (Baugh et al., 2002; Radstake et al., 2005). The *MIF* −173C allele has been associated with susceptibility to RA and with higher levels of radiological damage in RA patients (Radstake et al., 2005). Both high‐risk alleles (*MIF* −794CATT_7_ and −173C), as well as its 7C haplotype, have been correlated with increased *MIF* mRNA expression and MIF soluble levels (Bae & Lee, 2018; Baugh et al., 2002; Donn et al., 2002; Radstake et al., 2005; Yao et al., 2016). The *MIF* −173C allele may show greater sensitivity to clinical phenotype due to reduced locus heterogeneity at the −173 versus the −794 polymorphic sites.

In Mexico, there is only one study evaluating the *MIF* promoter polymorphisms in RA patients from Western Mexico (Llamas‐Covarrubias et al., 2013); therefore, there is little evidence to generalize the association between *MIF* gene and RA pathogenesis in this population. Considering the great genetic diversity in the Mexican population, the aim of this study was to evaluate the association of *MIF* polymorphisms (−794CATT_5‐8_ and −173G > C), circulating MIF levels and mRNA expression with RA susceptibility and disease activity in a population from Southern Mexico.

## 2 MATERIAL AND METHODS

### 2.1 Editorial policies and ethical considerations

Informed written consent was obtained from all patients and control subjects (CS) before enrolling into the study, according to the Declaration of Helsinki ethical guidelines. The investigation was approved by the Ethics Committee of Hospital General de Chilpancingo Dr. Raymundo Abarca Alarcon and by the Ethics, Investigation and Biosecurity Committee of the Centro Universitario de Ciencias de la Salud, Universidad de Guadalajara.

### 2.2 Subjects

This case–control study included 200 healthy subjects (control group) recruited from the general population and 200 RA patients classified according to the 2010 American College of Rheumatology/ European League Against Rheumatism criteria (Aletaha et al., 2010). Family history of RA/autoimmune disease was not considered as an exclusion criterion for the enrollment of the subjects. They were enrolled from the Rheumatology Department at Hospital General de Chilpancingo Dr. Raymundo Abarca Alarcon, Chilpancingo, Gro, Mexico. The disease activity was evaluated by the Disease Activity Score 28 (DAS28) (Prevoo et al., 1995). Both groups were Mestizo unrelated individuals from Southern Mexico with at least three generations of Mexican ancestry.

### 2.3 *MIF* polymorphisms −794CATT_5‐8,_ and −173G > C genotyping

The *MIF* −794CATT_5‐8_ polymorphism (*rs5844572*) was genotyped by conventional polymerase chain reaction (PCR) and polyacrylamide gel electrophoresis (PAGE), using the primers reported by Donn et al. (Donn et al., 2002), with the following PCR conditions: initial denaturalization at 95ºC for 4 min followed by 33 cycles at 95°C, 60°C and 72°C for 30 s at each temperature, and a final extension at 72°C for 2 min. The products obtained from the PCR were analyzed by PAGE on a 29:1 (12%) polyacrylamide gel at 100 V for 14 hr and stained with .02% AgNO_3_.

The *MIF* −173G > C polymorphism (*rs755622*) was analyzed by the PCR‐restriction fragment length polymorphism (PCR‐RFLP) technique, using the primers reported by Donn et al. (Donn et al., 2001) with the following PCR conditions: initial denaturalization at 95ºC for 4 min followed by 35 cycles at 95°C, 60°C and 72°C for 30 s at each temperature, and a final extension at 72°C for 2 min. The amplification products (366 bp fragment) from the PCR were verified by PAGE, followed by digestion with the restriction endonuclease enzyme *Alu I* (New England Biolabs) for 4 hr at 37ºC. Finally, the digested products were electrophoresed on a 29:1 (6%) polyacrylamide gel at 110 V for 2 hr and stained with .02% AgNO_3_. The G allele was represented in two fragments (268 and 98 bp) while the C allele resulted in 206, 98, and 62 bp fragments. Results were confirmed by automatized sequencing (Applied Biosystems) of randomly selected samples of each genotype for both polymorphisms.

### 2.4 *MIF* mRNA expression analysis

Total RNA was obtained from a randomly selected subset of 24 RA patients and 15 CS using TRIzol reagent (Invitrogen) according to the Chomczynski and Sacchi method (Chomczynski & Sacchi, 1987). RNA concentration and purity were verified by spectrophotometry (NanoDrop 2000c, Thermo Scientific). The cDNA synthesis was generated from 1 μg of total RNA by reverse transcription, using the oligo(dT)_15_ Primer (Promega Corporation) following the manufacturer protocol. *MIF* mRNA quantification was performed by real‐time PCR using UPL hydrolysis probes (catalog number 04,687,990,001, Roche Life Science). Primers and probes were obtained from the Universal ProbeLibrary System Assay Design program (Roche Life Science). Glyceraldehyde 3‐phosphate dehydrogenase (*GAPDH*) was used as a reference gene (UPL probe, catalog number 05,190,541,001, Roche Life Science). After validation of the reaction efficiency for both genes (*MIF* and *GAPDH*), PCR reactions were run in duplicate using the conditions indicated in the UPL Gene Expression Assay protocol in a LightCycler Nano System (Roche Life Science). *MIF* mRNA relative expression analysis was calculated with the 2^−ΔΔCq^ and 2^−ΔCq^ methods (Livak & Schmittgen, 2001).

### 2.5 MIF serum levels quantification

MIF serum levels were determined in 91 RA patients and 40 CS (randomly selected) by a commercial enzyme‐linked immunosorbent assay (ELISA) kit (LEGEND MAX Human Active MIF ELISA Kit, BioLegend), according to the manufacturer's instructions. MIF assay sensitivity was .0174 ± .0092 ng/ml.

### 2.6 Statistical analysis

Categorical variables were expressed as percentages and frequencies. The distribution of all continuous variables was examined by the Shapiro–Wilk normality test. Variables with a nonparametric distribution were expressed as median and 5–95th percentiles. Kruskal–Wallis test was used to analyze differences between three or more groups followed by Dunn's multiple comparison test. Meanwhile, Mann–Whitney U test was used to evaluate differences between two groups. Hardy–Weinberg equilibrium and comparisons of genotype, allele, and haplotype frequencies distributions between groups were evaluated with the χ^2^ test. Odds ratios (OR) and 95% confidence intervals (95% CI) were calculated to evaluate the risk association with RA. Linkage disequilibrium was evaluated with the SHEsis software. Statistical analysis was performed using the Stata Software version 11.1 and GraphPad Prism version 8.0.2. A *p* value < .05 was considered significant.

## 3 RESULTS

### 3.1 Characteristics of study subjects

Demographics and clinical characteristics of RA patients and CS are described in Table 1. RA patients had a median age of 46.5 (26.1–69) years and 94% of them were female. The average evolution time of the disease was 6 years, and the median of subjects had a moderate activity disease score according to the DAS28 index. Most patients were under combined conventional treatment with nonsteroidal anti‐inflammatory drugs (NSAIDs) (59%) and disease modifying antirheumatic drugs (DMARDs), mainly methotrexate (69.5%). The age and gender of the CS were like that of the RA patients. Fifty‐three (26.5%) RA patients reported a direct family history of RA, while this condition was present only in 23 (11.5%) individuals of the CS group (*p* < .0001).

**Table 1:** Clinical features of RA patients and control subjects

Parameter	RA (*n* = 200)	CS (*n* = 200)	*p*
Demographics			
Age, years^a^	46.5 (26.1–69)	47 (27.1–70)	.98
Gender			
Female/ Male^b^	94 (188)/ 6 (12)	94 (188)/ 6 (12)	1.0
Family history of RA^b^	26.5 (53)	11.5 (23)	**<.0001**
Clinical assessment			
Years of disease evolution^a^	6 (1–23)	—	—
DAS28^a^	3.2 (2.1–7.5)	—	—
Remission <2.6^b^	18.5 (37)	—	—
Low activity ≥2.6 to <3.2^b^	30.5 (61)	—	—
Moderate activity ≥3.2 to ≤5.1^b^	33 (66)	—	—
High activity >5.1^b^	18 (36)	—	—
WBC (10^3^/µL)^a^	6.68 (4.11–10.6)	6.6 (4.8–9.59)	.88
ESR (mm/h)^a^	34 (12–56)	27 (6.1–47)	**<.0001**
CRP (mg/dL)^a^	15.8 (3.3–104.1)	10.7 (2.5–46.5)	**<.0001**
RF (UI/mL)^a^	173.9 (3.1–300)	0 (0–15.1)	**<.0001**
Anti‐CCP (U/mL)^a^	107 (.1–900)	0 (0–5)	**<.0001**
Treatment			
NSAIDs^b^	59 (118)	—	—
Glucocorticoids^b^	41 (82)	—	—
DMARDs			
Methotrexate^b^	69.5 (139)	—	—
Hydroxychloroquine^b^	38.5 (77)	—	—
Sulfasalazine^b^	18 (36)	—	—
Leflunomide^b^	2.5 (5)	—	—

Abbreviations: Anti‐CCP, anti‐cyclic citrullinated peptide; CRP, C‐reactive protein; CS, control subjects; DAS28, disease activity score 28‐joint counts; DMARDs, disease modifying antirheumatic drugs. ESR, erythrocyte sedimentation rate; NSAIDs, nonsteroidal anti‐inflammatory drugs; RA, rheumatoid arthritis; RF, rheumatoid factor. Statistically significant data are shown in bold type.

a Data are expressed as median and (p5–p95).

b Data are expressed as the percentage and number of individuals (*n*).

### 3.2 Frequencies of alleles, genotypes, and haplotypes of *MIF* polymorphism

In order to address the association of *MIF* gene promoter polymorphisms with RA susceptibility, two distinct polymorphic regions including the −794CATT_5‐8_ (*rs5844572*) and −173G > C (*rs755622*) SNPs were investigated. The distribution of all genotypes and alleles was in Hardy–Weinberg equilibrium (HWE) and both polymorphisms were in linkage disequilibrium (D´ = .94, *data not shown*).

Regarding the −794CATT_5‐8_ polymorphism, the 6,7 genotype was the most frequent in RA (35%) and CS (32.5%) groups. On the other hand, the GC genotype of the −173G > C polymorphism was the most frequent in both study groups (RA 48% vs. CS 42%). In terms of haplotypes, in the RA population as well as in the controls we observed three predominant haplotypes (−794CATT_5‐8_/ −173G > C *MIF*): 5G (RA 13.5% vs. CS 11.2%), 6G (RA 46.5% vs. CS 50%), and 7C (RA 37.7% vs. CS 35.5%); the remainder of the haplotypes had very low frequencies (<1%) in both study groups.

The frequencies of alleles, genotypes, and haplotypes were very similar between both study groups, therefore, none of the two *MIF* polymorphisms evaluated were associated with RA risk (Table 2).

**Table 2:** Genotype, allele, and haplotype frequencies of −794CATT_5‐8_ and −173G > C *MIF* polymorphisms in RA patients and CS

Polymorphism	RA% (*n* = 200)	CS% (*n* = 200)	OR (95% CI)	*p*
−794CATT_5−8_ *MIF*				
Genotype				
5,5	0 (0)	2 (4)	—	—
5,6	16.5 (33)	12.5 (25)	1.67 (.83–3.37)	.12
5,7	11.5 (23)	8.5 (17)	1.71 (.76–3.84)	.15
6,6^a^	22.5 (45)	28.5 (57)	1	—
6.7	35 (70)	32.5 (65)	1.36 (.78–2.36)	.23
7,7	14.5 (29)	14.5 (29)	1.26 (.63–2.54)	.47
7,8	0 (0)	1.5 (3)	—	—
Allele				
5	14 (56)	12.5 (50)	1.18 (.75–1.86)	.44
6^a^	48.25 (193)	51 (204)	1	—
7	37.75 (151)	35.75 (143)	1.11 (.81–1.52)	.47
8	0 (0)	.75 (3)	—	—
−173G > C *MIF*				
Genotype				
GG^a^	36 (72)	40.5 (81)	1	—
GC	48 (96)	42 (84)	1.28 (.81–2.02)	.25
CC	16 (32)	17.5 (35)	1.02 (.55–1.90)	.92
Allele				
G^a^	60 (240)	61.5 (246)	1	—
C	40 (160)	38.5 (154)	1.06 (.79–1.42)	.66
Haplotype (−794CATT_5−8_/−173G > C *MIF*)				
5G	13.5 (54)	11.2 (45)	1.29 (.80–2.06)	.25
6G^a^	46.5 (186)	50 (200)	1	—
7C	37.7 (151)	35.5 (142)	1.14 (.83–1.56)	.38

Note

Haplotypes with a frequency <.03 were not included. Chi square test χ^2^; RA, rheumatoid arthritis; CS, control subjects; OR, odds ratio; CI, confidence interval.

a Reference category.

### 3.3 *MIF* polymorphisms and clinical characteristics of RA

**Figure 1:**
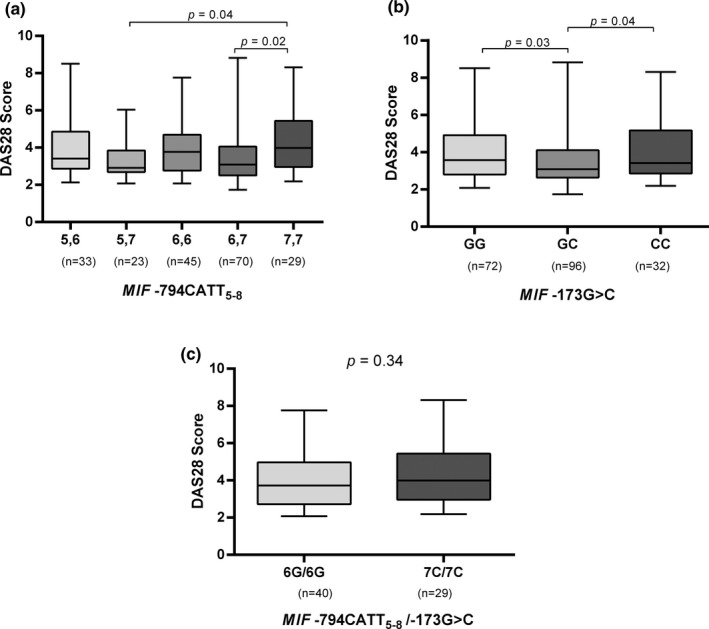
Disease activity (DAS28) according to the genotype distribution of MIF polymorphisms in RA. (a) Kruskal–Wallis test showed a significant difference in the DAS28 score according to MIF −794CATT5‐8 genotypes (*p* = .03); meanwhile, Dunn's multiple comparison test showed differences between the 7,7 and 5,7 genotypes (*p *=* *.04) and between 7,7 versus 6,7 genotypes (*p* = .02). (b) DAS28 score according to MIF −173G > C genotypes. (c) Haplotypes were inferred from homozygous subjects to MIF −794CATT5‐8 and −173G > C polymorphisms. Comparison among groups was performed using Mann–Whitney U test (b, c)

The relationship of *MIF* genotypes and haplotypes with the clinical characteristics of RA patients was also tested. We found a significant association of the two *MIF* polymorphisms (−794CATT_5‐8_ and −173G > C) with the DAS28 score. For the −794 CATT_5‐8_ polymorphism (Figure 1a), a significantly higher DAS28 score was observed in the carriers of the 7,7 genotypes in comparison with those carrying the 5,7 (*p* = .04) or 6,7 (*p* = .02) genotypes. Similarly, the −173 GG or −173 CC genotypes carriers had higher DAS28 score compared to GC genotype carriers (Figure 1b, *p* < .05): GG DAS28 = 3.5 (2.3–7.6); GC DAS28 = 3.08 (2.01–7.4); CC DAS28 = 3.42 (2.35–7.9). RA patients were also classified according to *MIF* haplotypes, but no association was found (Figure 1c, *p* = .34).

### 3.4 *MIF* serum levels and *MIF* mRNA expression in RA patients and CS

**Figure 2:**
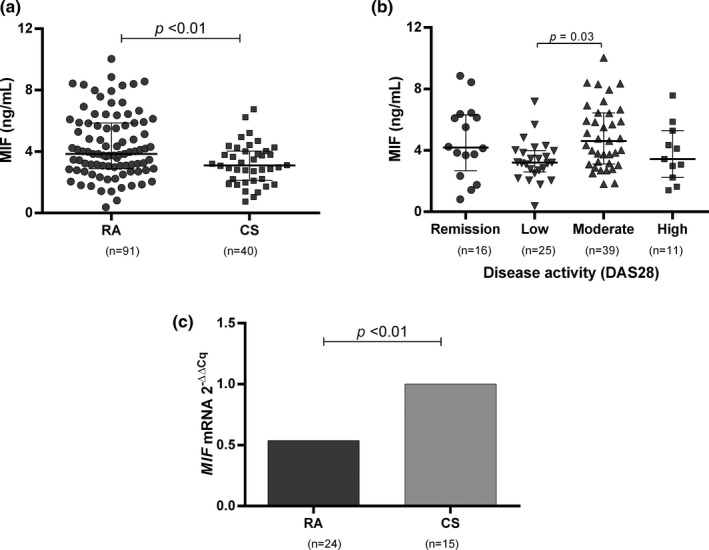
MIF serum levels and MIF mRNA expression. (a) Comparison of MIF serum levels between RA patients and CS. (b) MIF serum levels according to the disease activity score (DAS28) in RA patients. (c) MIF mRNA expression in RA and CS. Statistical analysis was performed using Mann–Whitney U test (a, c) and Dunn's multiple comparison test (b)

The MIF serum levels were higher in RA patients (3.8 ng/ml) than CS (3.1 ng/ml) (Figure 2a, *p* < .01). Additionally, a significant increase of MIF levels in RA patients with moderate disease activity (DAS28 ≥ 3.2 to ≤5.1) versus those with low disease activity (DAS28 ≥ 2.6 to <3.2) was found (Figure 2b, *p* = .03). On the other hand, the relative expression of *MIF* in total leucocytes from RA patients was 1.88‐fold lower (1/.53) than CS (Figure 2c, *p* < .01).

**Figure 3:**
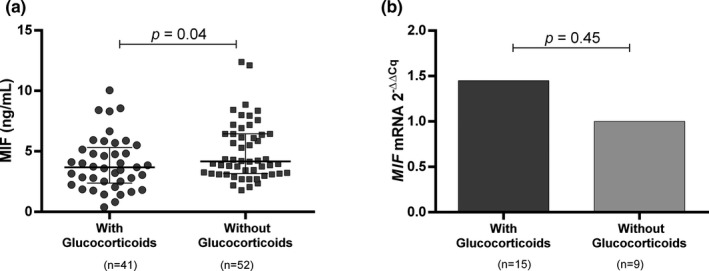
Effect of glucocorticoid treatment on MIF serum levels and MIF mRNA expression. (a) Comparison of MIF serum levels between RA patients with or without glucocorticoid treatment. (b) MIF mRNA expression between RA patients with and without glucocorticoid treatment. The medians were compared using the Mann–Whitney U test

The relationship between the MIF serum levels with the clinical parameters of RA patients was also evaluated but no significant differences were detected (*p* > .05, data not shown). However, we observed higher levels of MIF in those individuals who were not under glucocorticoid treatment (without glucocorticoids: median 2.36 ng/ml vs. with glucocorticoids: 2.15 ng/ml, Figure 3a, *p* = .04). This analysis according to treatment was also tested for the *MIF* mRNA expression; however, no association was found (Figure 3b, *p* = .45).

Finally, we evaluated the impact of the two *MIF* polymorphisms evaluated (−794CATT_5‐8_ and −173G > C) on the MIF serum levels and *MIF* mRNA expression. No significant differences in MIF serum levels or *MIF* mRNA expression between carriers of different alleles, genotypes, or haplotypes were observed in both study groups (data not shown).

## 4 DISCUSSION

RA is a complex disease with variable clinical manifestations and diverse physiopathological mechanisms; genetics and environment play a major role in the susceptibility to RA (Deane et al., 2017; Pratt, Isaacs, & Mattey, 2009). The heritability of RA is estimated to be 60%, which has been calculated from Finnish and English twin data (Kurkó et al., 2013; MacGregor et al., 2000). Thus, the genetic factors seem to have a major influence on the development of RA. These factors include the increased prevalence of RA within families (Deane et al., 2017), in accordance with a previous study in Western Mexico (Llamas‐Covarrubias et al., 2013), since we observed 26.5% of the patients with positive family history of RA, while this was only observed in 11.5% of CS (*p* < .0001).

Our working group has reported earlier the association of *MIF* promoter polymorphisms with the susceptibility to develop RA in Western Mexican population (Llamas‐Covarrubias et al., 2013), which is a Mestizo population with markedly European ancestry (60%–64%), followed by Amerindian (21%–25%) and African (≈15%) (Rangel‐Villalobos et al., 2008). However, due to great genetic diversity present in the Mexican population and given the necessity to consider ancestry patterns in different regions of Mexico for genetic susceptibility studies (Moreno‐Estrada et al., 2014), we performed this study in a Southern Mexican population, which is characterized by Amerindian ancestry (48%), followed by European (39%), Eurasian (10%), and African (4%) populations(Martínez‐Cortés et al., 2012).

Contrary to that observed in the Western Mexican population (Llamas‐Covarrubias et al., 2013), the genotypes of the studied *MIF* polymorphisms (−794 CATT_5‐8_ and −173G > C) were not associated with the predisposition to RA in the Southern Mexican population. In other populations, the *MIF* 7‐CATT and −173C alleles have been associated with inflammatory polyarthritis as well as radiologic damage in RA patients (Barton et al., 2003; Radstake et al., 2005). Conversely, the 5‐CATT allele has been linked to low disease severity in RA patients (Baugh et al., 2002). Most studies of *MIF* genetics have revealed its predominant role in autoimmune disease severity or clinical manifestations rather than in disease susceptibility, and differences observed in disease predisposition may reflect different effect sizes in genetically distinct populations.

In this Southern Mexican population, the *MIF* promoter polymorphisms were in linkage disequilibrium, similar to that reported for other populations (Barton et al., 2003; Donn et al., 2002; Martínez et al., 2007), including Western Mexico (Llamas‐Covarrubias et al., 2013). However, the haplotype frequencies obtained in each group were not associated with susceptibility to RA; this is consistent with the reported by Llamas–Covarrubias et al (Llamas‐Covarrubias et al., 2013). Meanwhile, there are other populations with an association of the haplotype 7‐CATT/‐173C to other autoimmune diseases like erythematosus systemic lupus (Sánchez et al., 2006), scleroderma (Wu et al., 2006), inflammatory polyarthritis (Barton et al., 2003), and idiopathic juvenile arthritis (Donn et al., 2004). Either this *MIF* haplotype does not have a major contribution in RA in Mexican populations or conclusions are limited by sample size.

The *MIF* promoter polymorphisms also were evaluated with respect to the DAS28 score for association with the disease activity of RA patients. The carriers of 7,7 (−794CATT_5‐8_) genotype had higher disease activity compared to 5,7 and 6,7 carriers. On the other hand, GC (−173G > C) genotype carriers showed less disease activity compared to carriers of other genotypes (GG or CC). In a previous research in Western Mexico, we identified and association of the 7‐CATT and −173*C alleles with high disease activity in RA patients (Llamas‐Covarrubias et al., 2013); thereby, seems that these *MIF* polymorphisms are not associated with the risk of developing RA, however, they may be associated with the activity of the disease. Nevertheless, the disease activity is highly modifiable by treatment and our patients were under a great diversity of pharmacological therapies, most of them with combined therapy (two or more DMARDs), thus the association between genotypes with disease activity should be taken with caution.

The increased MIF serum levels in RA patients and other inflammatory disorders has been previously reported (Jankauskas, Wong, Bucala, Djudjaj, & Boor, 2019), which agree with that observed in this study, as higher MIF serum levels were found in RA patients in comparison with the CS group. However, surprisingly, we observed 1.8‐fold less *MIF* mRNA expression in RA patients than in CS. This can be explained because the MIF serum levels are the products of different cell sources and it cannot be assured that the levels are unique products of white blood cells as tested in the *MIF* mRNA expression. Moreover, the discrepancy of mRNA and soluble levels could be explained by posttranscriptional level regulation. Keene JD has suggested that the ribonome (the total cellular complement of RNAs and their regulatory factors) is the major contributor to the discrepancies observed in the levels of mRNA‐protein, as the ribosomes modify mRNA processing, stability, and decay (Keene, 2001). It is further known that MIF is present in preformed intracellular stores and undergoes specialized regulation at the level of protein export (Merk et al., 2009). Posttranscriptional control also involves the participation of several noncoding RNAs with differential expression pattern in RA patients; most of these show a high expression level in joints and serum/plasma, such as miR‐16, miR‐21, miR‐24, miR‐223, and miR‐451 (Churov, Oleinik, & Knip, 2015). According to the miRDB database, three miRNAs have been reported with *MIF* (hsa‐miR‐451a, hsa‐miR‐629‐3p, hsa‐miR‐1537‐3p) being the target (Wong & Wang, 2015). In nonsmall cell lung cancer cases there is a negative correlation between *MIF* mRNA and hsa‐miR‐451a, and it is discussed that low expression of hsa‐miR‐451a is associated with a negative outcome of the disease (Goto et al., 2017). Specifically, miR‐451 is highly expressed in RA patients who were treated with methotrexate similar to the newly diagnosed RA free of DMARDs subjects; the miR‐451 levels also positively correlate with DAS28 score and ESR (Smigielska‐Czepiel et al., 2014). According to the previously published information, we hypothesize that there may exist an upregulation of miR‐451 in our RA patients, which in turn could be diminishing the *MIF* mRNA but the translational rate is not affected.

The most critical functions of MIF in the serum of RA patients could be the regulation of macrophage and lymphocyte activation or stimulation of the synthesis of other proinflammatory mediators; thus, MIF serum levels could be associated with disease activity. We found significant differences in MIF serum levels between patients with different disease activity measured by the DAS28 score. Mainly, it was observed that patients with a moderate activity of the disease had higher MIF levels compared to patients with low activity. There was also a trend toward lower levels of circulating MIF in patients with high activity compared to the other study groups that was not statistically significant. These discrepancies could be explained by the treatment of the different groups, as patients with high activity generally have higher doses of immunosuppressive drugs, such as glucocorticoids. Lower MIF serum levels were observed for instance in RA patients under glucocorticoid treatment, although *MIF* mRNA expression was not affected by glucocorticoids. The effects of glucocorticoids on *MIF* regulation are cell‐ and dose‐specific (Alourfi et al., 2005). Very low concentrations of glucocorticoids induce MIF secretion while mRNA levels remain unchanged (Calandra et al., 1995; Leng et al., 2009), and it is suggested the glucocorticoids may act to increase MIF secretion by posttranscriptional mechanisms (Flaster, Bernhagen, Calandra, & Bucala, 2007; Petrovsky et al., 2003).

Finally, we compared the *MIF* mRNA expression and the MIF serum levels according to both *MIF* polymorphisms to examine the effect of these genetic variants on *MIF* gene regulation; this analysis showed no association between any of the alleles, genotypes, or haplotypes and the MIF serum levels or the amount of mRNA in total leukocytes of patients with RA or CS. This agrees with previous studies from Western (De la Cruz‐Mosso et al., 2014; Llamas‐Covarrubias et al., 2013; Morales‐Zambrano et al., 2014) and Southern Mexican population (Matia‐García et al., 2015); nevertheless, studies in European, Caucasian, and African American populations have reported that *MIF* promoter polymorphisms affect MIF serum levels (Radstake et al., 2005; Sreih et al., 2011). More studies are required on this topic to clarify these discrepancies between populations. Further studies should focus on the functional effect of these variants on the binding of specific transcriptional factors in a cell‐dependent manner.

In conclusion, *MIF* −794CATT_5‐8_ and −173G > C polymorphisms are not susceptibility markers for RA in the Southern Mexican population. However, the 7,7 (−794CATT_5‐8_) and GC (−173G > C) genotypes are associated with high and low disease activity, respectively. Moreover, higher MIF serum levels are associated with moderate disease activity in RA patients. Therefore, we suggest a prominent role of MIF in RA disease activity.

## CONFLICT OF INTEREST

No conflict of interest was declared by the authors.
